# A novel one-step expression and immobilization method for the production of biocatalytic preparations

**DOI:** 10.1186/s12934-015-0371-9

**Published:** 2015-11-14

**Authors:** Ilka Sührer, Timo Langemann, Werner Lubitz, Dirk Weuster-Botz, Kathrin Castiglione

**Affiliations:** Institute of Biochemical Engineering, Technische Universität München, Boltzmannstr. 15, 85748 Garching, Germany; BIRD-C GmbH & Co KG, Erne-Seder-Gasse 4/2, 1030 Vienna, Austria

**Keywords:** Immobilization, Phage PhiX174, Membrane anchor, β-galactosidase, Cytochrome b_5_

## Abstract

**Background:**

Whole cell biocatalysts and isolated enzymes are considered as state of the art in biocatalytic preparations for industrial applications. Whole cells as biocatalysts are disadvantageous if substrate or products are toxic to the cells or undesired byproducts are formed due to the cellular metabolism. The use of isolated enzymes in comparison is more expensive due to the required downstream processing. Immobilization of enzymes after purification increases preparation costs for biocatalysts significantly, but allows for the efficient reuse of the enzymes in the biocatalytic process. For a more rapid processing one-step expression and immobilization is desirable.

**Results:**

This study focused on the development of a new one-step expression and immobilization technique for enzymes on the example of the β-galactosidase from *Escherichia coli* K12. The enzyme was expressed in *E. coli* with a C-terminal membrane anchor originating from cytochrome b_5_ from rabbit liver and was thus in situ immobilized to the inner surface of the cytosolic membrane. Then, the expression of a lytic phage protein (gene E from PhiX174) caused the formation of a pore in the cell wall of *E. coli*, which resulted in release of the cytosol. The cellular envelopes with immobilized enzymes were retained. Batch and fed-batch processes were developed for efficient production of these biocatalysts. It was possible to obtain cellular envelopes with up to 27,200 ± 10,460 immobilized enzyme molecules per cellular envelope (753 ± 190 U/g_dry weight_). A thorough characterization of the effects of membrane immobilization was performed. Comparison to whole cells showed that mass transfer limitation was reduced in cellular envelopes due to the pore formation.

**Conclusion:**

In this study the feasibility of a new one-step expression and immobilization technique for the generation of biocatalytic preparations was demonstrated. The technique could be a useful tool especially for enzyme systems, which are not suitable for whole-cell biocatalysts due to severe mass transfer limitations or undesired side reactions mediated by cytosolic enzymes.

**Electronic supplementary material:**

The online version of this article (doi:10.1186/s12934-015-0371-9) contains supplementary material, which is available to authorized users.

## Background

The application of whole cells and isolated enzymes are state-of-the-art techniques for industrial processes. Whole cell biocatalysts are cheap in production and have internal cofactor regeneration [1]. However, they can produce undesired byproducts due to the cellular metabolism, which complicate downstream processing [2]. Moreover, the cell wall can cause mass transfer limitation and toxic substrates or byproducts can be greatly damaging to the whole cell biocatalyst [1–4]. Therefore, enzymes are frequently purified. This does, of course, prevent the formation of undesired byproducts and thereby greatly facilitates the downstream processing [5]. However, the purification of enzymes is expensive in time and costs, causes losses during the purification and possibly a reduction in the catalytic activity [6, 7]. Immobilization of enzymes after purification increases preparation costs for biocatalysts, but allows for the efficient reuse of the enzymes in the biocatalytic process [5, 8]. The majority of immobilization techniques either use artificial surfaces to attach or encapsulate the catalyst in artificial particles [8]. Furthermore, purification is required prior to the immobilization, which is time consuming and cost intensive. An immobilization technique, which offers one-step expression and immobilization is therefore desirable. The “outer surface- or auto-display” of enzymes on the outer membrane of the cells is one studied option [4, 9, 10]. Here, the enzymes are attached to the outer membrane by fusing them to outer membrane proteins or so called autotransporters, to ensure sufficient translocation of the enzymes to the outer membrane [9–11]. Moreover, such whole cell biocatalysts displaying enzymes on their surface retain their complete metabolism, which can again cause the formation of undesired byproducts. Therefore, a new approach of one-step expression and immobilization was aimed for.

A new option studied in this work is immobilization of the desired enzyme or enzyme system to the inner membrane of *Escherichia* *coli* (*E.* *coli*). Biocatalysts are then created as cellular envelopes which contain afore immobilized enzymes by the removal of the cytosol. The cytosol can be removed by the formation of a pore by the lytic protein E from phage PhiX174. Protein E is a 91 amino acid long protein which lacks catalytic activity [12]. It inserts into the inner membrane of gram-negative cells. Subsequently, a conformational change takes place which eventually leads to fusion of inner and outer membrane due to protein E. A lysis pore (40–200 nm) is formed, the cytosol is released due to the osmotic pressure and a cellular envelope is retained. The cellular envelopes allow harvesting and treatment similar to whole cells [13–15]. If proteins with membrane anchors were expressed prior to lysis, the cellular envelope will contain afore immobilized enzymes [16]. Generally, the lytic phage protein E can be used to create cellular envelopes from gram negative bacteria and has a wide variety of applications [14, 17]. So far most studies focused on the generation of vaccines from pathogens. First experiments on immobilization within the cellular envelopes regarded additional antigens to enhance immune response, which were expressed in low amounts [16].

**Fig. 1:**
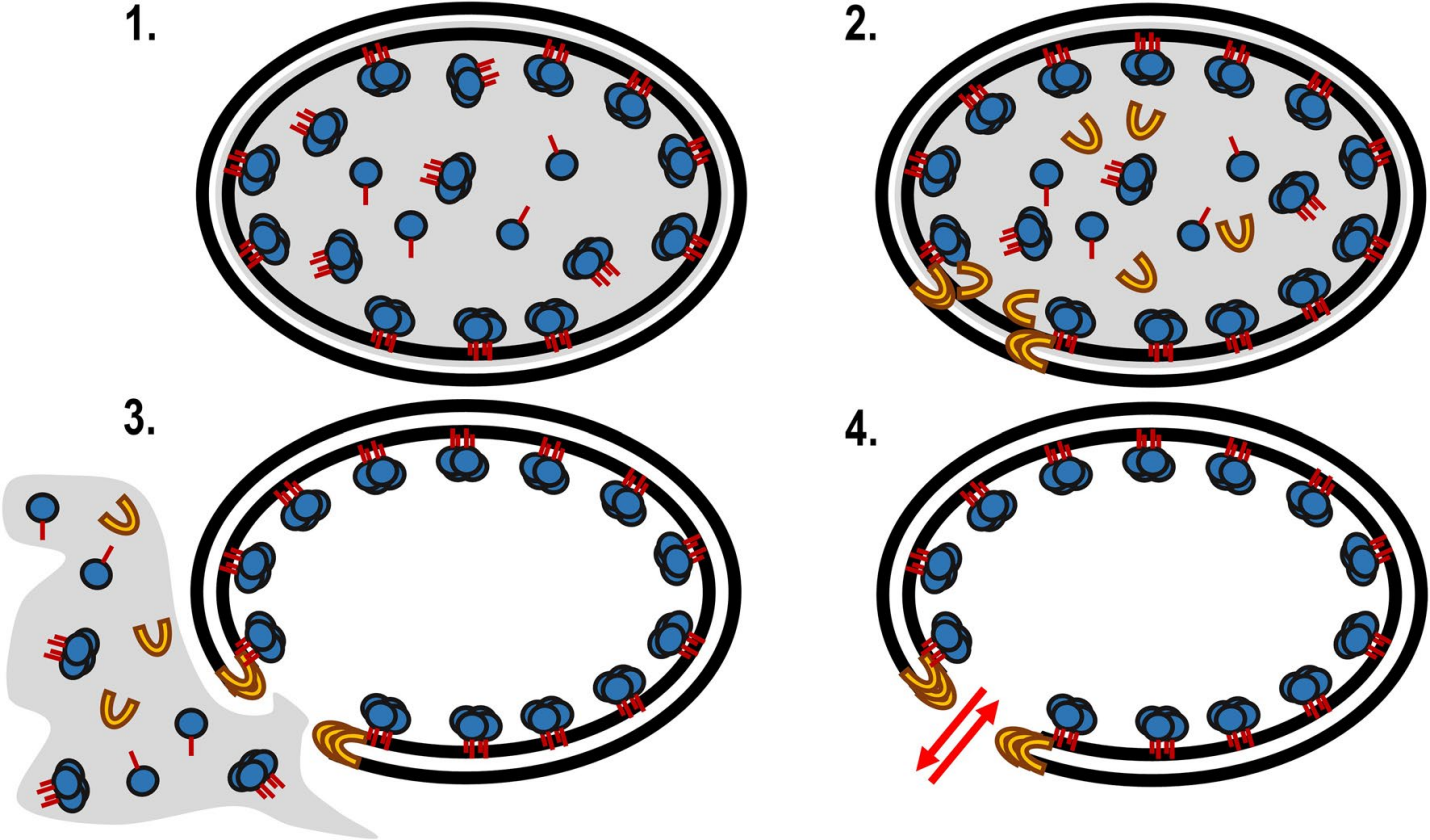
Schematic display of the new technique using E mediated lysis. *1* Expression of β-galactosidase as a model enzyme with C-terminal membrane anchor from cytochrome b_5_ (rabbit liver), posttranslational tetramer formation and inner membrane insertion. *2* Expression of lytic phage protein E and insertion into the cell membranes. *3* Pore formation by protein E and lysis with release of the cytosol. *4* Cellular envelope with immobilized enzymes and lysis pore

This paper is focused on the efficient preparation of *E. coli* cell envelopes with large amounts of immobilized enzymes at the inner surface of the cytosolic membrane. A schematic description is given in Fig. 1. β-Galactosidase from *E.* *coli* K12 was chosen to be immobilized as a model enzyme using the C-terminal hydrophobic sequence from cytochrome b_5_ from rabbit liver. This fusion protein has been described to insert spontaneously into membranes and liposomes [18]. C-terminal membrane anchoring is assumed to occur posttranslationally [18, 19]. So the new technique was independent from signaling peptides and trans-membrane channeling.

## Results and discussion

### Production of cellular envelopes with immobilized β-galactosidase

**Fig. 2:**
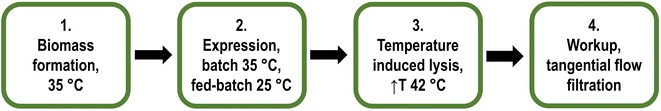
Schematic display of the production process. Expression in batch and fed-batch processes was chemically induced using 0.1 mM IPTG. Biomass formation was performed as a batch process in all experiments

In order to evaluate the new system, the β-galactosidase from *E.* *coli* K12 was fused with the C-terminal hydrophobic sequence of cytochrome b_5_ from rabbit liver, resulting in the β-gal-cyt b_5_-fusion protein [18]. So far, research regarding phage PhiX174 protein E mediated lysis focused on generating various vaccines, which do not require high expression levels of the antigen [16]. In this work biocatalyst were aimed for, which consequently need high numbers of enzyme molecules to provide sufficient catalytic activity. High expression levels of membrane proteins are often damaging to the cells [20, 21]. As the cellular vitality is crucial for protein E mediated lysis [12, 22], a low to medium copy number plasmid (pCOLADuet^®^) and a high copy number plasmid (pET28a) were compared. Cultivation in a stirred tank reactor was used to validate industrial applicability and to ensure cellular vitality by supply with sufficient amounts of dissolved oxygen and nutrients. Batch and fed-batch processes were compared to find the best expression conditions. In all experiments *E.* *coli* C41 (DE3) cells were used as they are better able to maintain cellular vitality despite membrane protein overexpression [20]. Cells were grown and subsequently chemically induced with isopropyl β-d-1-thiogalactopyranoside (IPTG). Lysis by protein E expression was controlled using the temperature sensitive promotor cI857 and lysis was induced by a shift to 42 °C. Cellular envelopes were washed using tangential flow filtration and analyzed for biocatalytic activity. A schematic description of the process is given in Fig. 2.

**Fig. 3:**
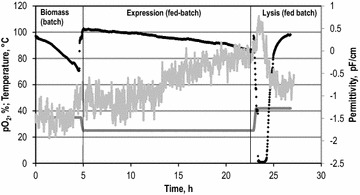
Exemplary online data from a fed-batch process. The dissolved oxygen (*black*), the temperature (*dark gray*) and the permittivity (*light gray*) are displayed as function of process time. The three phases of the experiment, biomass formation, expression and lysis are indicated

It was possible to obtain successful overexpression and lysis in all experimental setups with IPTG concentrations ≤0.1 mM, regardless if batch or fed-batch mode was used. Higher IPTG concentrations rendered the cells unfit for E mediated lysis. All processes with respective conditions offered lysis yields of >99.0 %. Notably, all obtained cellular envelopes exhibited catalytic activity. So, it was possible to generate new biocatalysts consisting of the cellular envelope with membrane bound β-gal-cyt b_5_. Cultivation in a stirred tank reactor was successfully used for the production, indicating feasibility for industrial applications. The main process parameters and results are summarized in Table 1. Additionally, permittivity measurements with a biomass probe were used for the observation of E mediated lysis. The polarizability and thus the permittivity of a cell culture can be correlated to biomass concentration, the higher the polarizability, the more cells and the higher the biomass concentration [23]. It was possible to detect E mediated lysis as the decline in permittivity caused by the loss in membrane potential of the cells. Figure 3 shows exemplary data from a fed-batch process. As depicted, the permittivity increases with cellular proliferation. During lysis a sudden decrease can be observed caused by the disruption of the cell and loss of the cytosol. The data also coincide with the amount of dissolved oxygen which increases as lysed cells no longer require oxygen. The increase in dissolved oxygen along with a decrease of the optical density is an indicator for E mediated lysis [14]. Notably, the amount of dissolved oxygen also increases if cells die due to low cellular vitality, while permittivity is largely maintained in that case (data not shown). So, the observation of permittivity offers a new tool for the observation of E mediated lysis on production scale. The cellular envelopes gained under different conditions (batch and fed-batch process) containing the immobilized β-gal-cyt b_5_ were washed using tangential flow filtration and analyzed for enzyme activity. Lyophilized cellular envelopes were stained with RH414 and the corresponding positive population was detected using flow cytometry. The dye RH414 binds to membranes and can be used to stain cells and cellular envelopes to remove background noise caused by other particles for a more accurate quantification [24]. The data was used to obtain a dry weight to particle concentration correlation (see Additional file 1: Figure S1). According to the correlation, the amount of activity per dry weight of cellular envelopes was calculated. The results for all different experimental setups are compared in Table 1. Both batch processes yielded more than twofold higher activities per dry weight than those obtained in fed-batch cultivation with up to 753 ± 190 U/g_dry weight_. Compared to the fed-batch process the activity per dry weight in both batch processes was higher, however the final cell dry weight (CDW) concentration was bisected. So the total activity obtained in all processes was roughly the same. As these were first approaches, the increase of final CDW concentrations prior to lysis should be a target of future experiments. As the maximum amount of enzymes per cellular envelope was of interest, all further experiments employed cellular envelopes that were produced in batch mode with the pET28a vector. The new biocatalysts were characterized regarding the amount of immobilized enzyme molecules per cellular envelope, the effect of the immobilization on the enzyme activity and alterations in mass transfer limitation.

**Table 1:** Summary of process parameters and results

	Plasmid	Expression duration, h	Expression temperature, °C	CDW concentration prior to lysis, g/L	Activity per dry weight cellular envelopes, U/g
Fed-batch	pCOLADuet	18	25	8.5 ± 0.3	312 ± 29
Batch	pCOLADuet	3	35	4.3 ± 0.3	632 ± 250
Batch	pET28a	3	35	3.9 ± 0.2	753 ± 190

Expression was chemically induced with 0.1 mM IPTG after biomass formation in a batch phase at 35 °C

*CDW* cell dry weight

### Characterization of the new biocatalyst

First, the number of immobilized enzyme molecules was determined. Therefore, a sandwich enzyme linked immunosorbent assay (ELISA) was established. Purified β-galactosidase was used to calculate the corresponding amount of β-gal-cyt b_5_ molecules within one cellular envelope. The cellular envelopes were disrupted using sonication and detergent prior to the application to the assay in order to solubilize the immobilized enzymes. However, the applied method did not enable a complete solubilization of all membrane bound enzymes. Therefore, the result was corrected according to the residual activity detected in the debris after disruption. Notably, the application of the detergent did not have an effect on enzyme activity (see Additional file 2: Figure S2). Multiple batch processes with pET28a vector were analyzed. Using sandwich ELISA quantification the amount of membrane bound β-gal-cyt b_5_ molecules in this first attempt was determined already to be 27,200 ± 10,460 per *E. coli* cell envelope. The highest amount of immobilized enzyme molecules on the outer membrane was achieved using a P450 enzyme with up to 180,000 molecules per *E. coli* cell [11, 25]. Generally, the number of immobilized molecules using surface display ranges between 15,000 and 180,0000 [25]. So, the number of molecules displayed on bacterial surfaces is depending on the kind of enzyme used and the new system can assumed to be generally in the same order of magnitude as the outer membrane system. Therefore, the establishment of the new one-step immobilization and expression technique for new biocatalysts was successful.

**Fig. 4:**
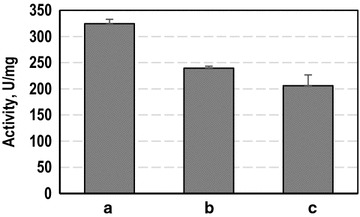
Comparison of activities of free and membrane bound β-gal-cyt b_5_. The activity of β-gal-cyt b_5_ immobilized to SUV (**a**) and the activity of the same samples after solubilization of β-gal-cyt b_5_ by detergent treatment (**b**) are compared. The reference activity (**c**) was obtained from purified β-galactosidase with N-terminal His_6_-tag

For a thorough characterization of the new technique, the effect of membrane immobilization was determined. The solubilization of β-gal-cyt b_5_ from cellular envelopes was incomplete and for a characterization of the β-gal-cyt b_5_ a complete solubilization was required. β-gal-cyt b_5_ is known to insert spontaneously to artificial and cellular membranes [18, 19]. Therefore, β-gal-cyt b_5_ was immobilized to artificial liposomes which could be easily degraded by detergents. Liposomes were generated as small unilamellar vesicles (SUV). The cleared lysate from cells overexpressing β-gal-cyt b_5_ was applied to the liposomes. It contained soluble β-gal-cyt b_5_ molecules that were not inserted into the plasma membrane. It was possible to immobilize β-gal-cyt b_5_ to artificial SUV from crude protein extracts. Notably, as only crude extract was used, other *E.* *coli* host cell proteins also immobilized to the artificial liposomes (see Additional file 3: Figure S3). The obtained liposomes contained sufficient activity for a characterization of the immobilized β-gal-cyt b_5_. The artificial SUV were analyzed in activity assays and subsequently disrupted by detergent. Sandwich ELISA was used to quantify the number of enzyme molecules bound to the liposomes. Using the activity prior to disruption and the amount of β-gal-cyt b_5_ detected using ELISA, the activity of the membrane bound molecules was calculated. The calculated activity of membrane bound and soluble β-gal-cyt b_5_ were compared to a reference activity gained from purified β-galactosidase with N-terminal His_6_–tag using the same conditions. The results are summarized in Fig. 4. The activity of membrane bound β-gal-cyt b_5_ was 324 ± 9 U/mg, whereas the free β-gal-cyt b_5_ had an activity of 239 ± 4 U/mg. So, the activity was increased by 35.5 ± 7.8 % due to the immobilization on the membrane. Notably, the reference activity determined using purified β-galactosidase with N-terminal His_6_-tag was 206 ± 20 U/mg and consequently lower than the activity of soluble β-gal-cyt b_5_. However, taking the standard deviation into account it was only marginally smaller. Generally, the activity was enhanced by immobilization rather than decreased. Immobilization of enzymes can have multiple effects which result in altered enzyme properties [26, 27]. As the β-galactosidase is a tetramer, one possible explanation could be the stabilization of the tetramer in membrane bound multimers. If multimeric proteins are not connected colvalently, e.g. by disulfide bonds, they can be prone to dissociation. The rate constants of association and dissociation then reduce the catalytic activity of the multimer [26]. It has been demonstrated, that attachment of multimers to surfaces can prevent dissociation of the subunits. Consequently the multimer is stabilized and enzyme performance enhanced compared to the unbound enzymes [26, 27]. A respective stabilization can be assumed a possible explanation for the increased activities of membrane bound β-gal-cyt b_5_ compared to the free enzyme. Moreover, the data concurs with data from George et al., who also describe an increased activity of the membrane bound fusion compared to the soluble enzyme [18].

### Analysis of mass transfer limitation

**Fig. 5:**
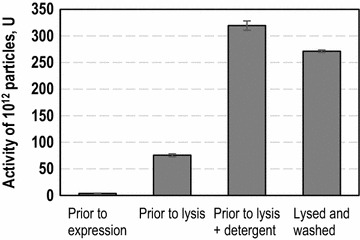
Comparison of β-gal-cyt b_5_ activity in whole cells and cellular envelopes for the analysis of mass transfer limitation. The β-gal-cyt b_5_ activity was compared in whole cells prior to expression, whole cells prior to lysis, whole cells treated with detergent and cellular envelopes after workup. All samples were adjusted to equivalent particle concentrations using RH414 staining in flow cytometry

One issue frequently faced when using immobilized enzymes and whole cell biocatalysts is mass transfer limitation of substrates or co-factors [1–4]. Therefore, mass transfer was analyzed in cellular envelopes with membrane bound β-gal-cyt b_5_ compared to whole cells. Activity assays were used to compare cellular envelopes and whole cells sampled after expression but prior to lysis. Additionally, whole cells taken prior to lysis were treated with detergent in order to overcome mass transfer limitation. Exemplary data is given in Fig. 5. As demonstrated, cells prior to lysis had a reduced activity compared to cellular envelopes. So, due to the formation of the pore mass transfer limitation was reduced. The activity of cells treated with detergent was assumed as 100 %. As the detergent treatment had no effect on enzyme activity (see Additional file 3: Figure S3) it equaled the maximum possible activity with no mass transfer limitation due to the cell wall. Cellular envelopes contained 78 ± 13 % activity and cells prior to lysis 26 ± 10 %, respectively. Notably, not all activity is retained in cellular envelopes after lysis. As described above, C-terminal membrane immobilization is assumed to occur posttranslationally and is also incomplete [18, 19]. So, the difference in activity of cellular envelopes can be explained by the loss of soluble enzyme due to loss of the cytosol during lysis. The amount of membrane bound activity concurs with data described by George et al., who reported up to 80 % of immobilized enzyme activity of a similar β-gal-cyt b_5_ enzyme [18]. It was possible to demonstrate a reduction in mass transfer limitation due to the formation of the lysis pore. The new biocatalysts thus proved better catalysts than whole cells prior to lysis regarding this application.

## Conclusion

A new one-step expression and immobilization system was established for the production of cellular envelopes with immobilized enzymes as new biocatalytic preparations. Batch and fed-batch cultivation of *E. coli* in a stirred tank reactor were used to produce cellular envelopes by protein E mediated lysis, which contain afore immobilized fusion protein β-gal-cyt b_5_ attached to the inner surface of the cytosolic membrane. Permittivity measurements were used for the detection of protein E mediated lysis. It was possible to obtain cellular envelopes which contain 27,200 ± 10,460 β-gal-cyt b_5_ molecules with an activity of 753 ± 190 U/g_dry weight_. A thorough characterization of the effects of membrane immobilization was possible. By immobilizing the β-gal-cyt b_5_ to artificial liposomes, the effect of membrane immobilization was characterized. Notably, the activity was increased by 35.5 ± 7.8 % after immobilization to the membrane compared to the soluble β-gal-cyt b_5_. This observation can be explained by the stabilization of the β-gal-cyt b_5_-tetramer in the membrane. Additionally, mass transfer limitation was analyzed. It was demonstrated that mass transfer limitation is reduced due to the pore in the cell envelope formed due to protein E mediated lysis. So, the cellular envelopes proved advantageous compared to whole cells prior to lysis. A new tool for the production of immobilized enzymes as biocatalytic preparations was established. The new technique offers an alternative for whole-cell biotransformations which are limited by mass transfer limitation or rendered inapplicable by the formation of undesired byproducts due to host cell metabolism.

## Methods

### Chemicals

All chemicals were purchased from various suppliers in analytical grade and were used without further purification.

### Cloning

All primers were purchased at Eurofins MWG Operon (Ebersberg, Germany) unless stated otherwise. The β-galactosidase with cytochrome b_5_ membrane anchor was cloned in allusion to George et al. [18]. The β-galactosidase was amplified from genomic DNA from *E. coli* K12 using primers eliminating the stop codon and cloned into the pCOLADuet^®^ (Novagen^®^, Merck KGaA, Darmstadt, Germany) vector via NcoI and AscI. The oligonucleotides for the cytochrome b_5_ membrane anchor were purchased from biomers.net (Ulm, Germany) as 5′-phsophorylated single strands containing a stop codon (see Additional file 4: Table S1). The single strands were prepared by heating the solution to 95 °C for 5 min. After alignment for 30 min at 65 °C, they were cloned to the C-terminus of the β-galactosidase via AscI and NotI. For cloning of the fusion protein β-gal-cyt b_5_ to the pET28a vector (Novagen^®^, Merck KGaA,Darmstadt, Germany), the DNA was amplified and inserted into the vector via NheI and XhoI. For expression and purification of the β-galactosidase without membrane anchor, the corresponding gene was amplified with N-terminal His_6_-tag from genomic DNA and cloned to pET28a via NcoI and XhoI.

Standard methods were used for PCR, ligation, transformation and plasmid preparation as described in Sambrook and Russel [28].

The pGlysivb vector containing the lysis gene E from PhiX174 controlled by the temperature sensitive promotor cI857 is described in Jechlinger et al. [29].

### Microorganisms

Cloning was conducted using *E.* *coli* DH5α cells (Invitrogen, Carlsbad, USA). All expressions were performed with *E.* *coli* C41 (DE3) cells (Lucigen^®^ Corporation, Middleton WI, USA) containing pGlysivb and pCOLADuet^®^ or pET28a with β-gal-cyt b_5_.

### Media

For long term storage and determination of colony forming units the cells were kept on Plate Count Agar (Carl Roth, Karlsruhe, Germany). A defined medium according to Delisa et al. [30] was used for cultivation in the stirred-tank reactor and the corresponding precultures in shaking flasks. In all other cases LB medium was used. In batch processes the initial glucose concentration was 20 g/L. In fed-batch processes the initial glucose concentration was 5 g/L during biomass formation. During feeding the growth rate was adjusted using a modified feeding medium containing 50–100 g/L glucose, 5 g/L antifoam 204 (Sigma-Aldrich, München, Germany), 2.5 g/L MgSO_4_, 5 mg/L Fe(III) citrate, 1.63 mg/L ethylenediaminetetraacetic acid (EDTA), 2 mg/L Zn(CH_3_COO)_2_, 2.5 mg/L CoCl_2_, 15 mg/L MnCl_2_, 3 mg/L H_3_BO_3_ and 2.5 mg/L Na_2_MoO_4_. 10 µg/mL gentamycin and/or 30 µg/mL kanamycin were added as required for selection.

### Cultivation in a stirred tank reactor

For precultures a single colony was used to inoculate 4 mL LB. 1 mL cells were subcultured and used to inoculate an unbaffled shaking flask containing defined medium at 20 % nominal volume. After incubation at 30 °C, 200 rpm overnight, the cells were harvested, concentrated to 1/5 of the initial volume and used for inoculation.

Batch and fed-batch cultivations were performed in a 3.6 L stirred-tank reactor (Infors-HT, Bottmingen, Switzerland), which was equipped with two Rushton turbine impellers. Cells from precultures were used to inoculate 1.5 L medium to an initial optical density at 600 nm (OD_600_) of 0.5. The pH was adjusted to 7.2 using 12.5 % (v/v) NH_4_OH and 1 M H_3_PO_4_. The dissolved oxygen was set to >20 % pO_2_ air saturation by adjusting the aeration up to 8 L/min, the stirrer was set to 1000 rpm. The permittivity was observed using a biomass probe (I biomass, HAMILTON Bonaduz AG, Bonaduz, Swizerland). In batch processes the temperature was 35 °C during biomass formation and expression. In fed-batch processes the temperature was 35 °C during biomass formation and 25 °C during expression. In batch and fed-batch processes expression was induced using 0.1 mm IPTG. For batch processes the cells were induced at OD_600_ ~ 1.5 to ensure sufficient amounts of glucose during lysis. The expression lasted 3 h at 35 °C. In fed-batch cultivations, the expression was induced upon glucose depletion and lasted 18 h at 25 °C. Feeding was initiated in fed-batch cultivations upon glucose depletion. An exponential growth was adjusted during expression with µ_s_ = 0.075 1/h according to Jenzsch et al. [31]. During lysis in fed-batch processes the growth rate was increased to µ_s_ = 0.3 1/h until glucose accumulation was detected. At this point the feeding was stopped. The process was terminated in both batch and fed-batch mode by initiating E mediated lysis using a temperature shift to 42 °C, as described by Langemann et al. [14]. When the decline in OD_600_ and increase in dissolved oxygen (pO_2_) indicated lysis, 50 µg/mL ampicillin were added.

### Workup of cellular envelopes

Concentration and washing was achieved by tangential flow filtration according to Langemann et al. [14] using a 0.22 µm hollow fiber module with 4.2 m^2^ surface. After lysis the cellular envelopes were concentrated to 1/3 of the initial volume. Then, the cellular envelopes were frozen at −20 °C and subsequently washed three times using phosphate buffered saline (PBS), pH 7.4 with 1:3 dilution in each step. The cellular envelopes were stored at −20 °C.

### Colony forming units

Colony forming units (cfu) were used to quantify the lysis yield. Samples were diluted accordingly in sterile 0.85 % (w/v) NaCl and spread on agar plates containing no antibiotics. After breeding at 30 °C overnight, the lysis yield was determined by counting the cfu.

### Protein expression

Expression and purification of β-galactosidase with C-terminal His_6_–tag and gal-cyt b_5_ for liposome formation was performed in unbaffled shaking flasks with LB medium at 20 % nominal volume, which were inoculated from a single colony. Expression and purification of the β-galactosidase with C-terminal His_6_-Tag was performed as described in Sührer et al. [32]. For expression of β-gal-cytb_5_ for liposome experiments, the cells were grown until OD_600_ was 0.6 and induced with 1 mM IPTG. Expression lasted for 3 h at 35 °C. Subsequently, the cells were harvested and PBS containing 1 mM phenylmethanesulfonylfluoride (PMSF) was added at a ratio of 5 mL per g wet pellet weight. Then, the cells were disrupted using a 2 mm tip sonicator for 10 min with an amplitude of 126.5 µm and the lysate was cleared for 30 min at 50,000x*g*. Soluble membranes were removed from the clear supernatant by ultracentrifugation for 1 h, 125,000×*g* at 4 °C. Subsequently, the soluble protein was concentrated to 1/5 of the initial volume using 3 kDa molecular weight cut off (MWCO) Amicon Ultra-15 spin columns (Merck Millipore, Darmstadt, Germany) at 4500×*g*.

### ELISA

Prior to application, samples were treated with Popculture^®^ detergent (Merck Millipore, Darmstadt, Germany) according to manufacturer’s instructions. Cellular envelopes were additionally disrupted with a 2 mm tip sonicator for 10 min with an amplitude of 126.5 µm for 10 min and subsequently harvested (30 min, 50,000×*g*). Then, the protein concentration was determined in samples from cellular envelopes using the Pierce^®^ bicinchonic acid (BCA) protein assay kit (Thermo Scientific, Schwerte, Germany) according to manufacturer’s instructions and the samples were diluted to 20 µg/mL. Liposomes were treated with detergent only and applied without centrifugation using dilutions of 1:20–1:60. All samples were diluted in PBS, pH 7.4. A standard sandwich ELISA protocol was used according to http://www.abcam.com/ (accessed in November 2014). Sandwich ELISA was performed using Maxisorp^®^ Plates (Nunc^®^, Roskilde, Denmark). The capture antibody (monoclonal anti β-galactosidase from mouse, MA1-152, Thermo Scientific, Schwerte, Germany) was applied at a 1:2000 dilution in coating buffer (0.1 M sodium carbonate, pH 9.6) for 2 h at room temperature (RT). 5 % (w/v) milk powder in PBS (blocking buffer) was used for blocking for 2 h at RT. Then, 100 µL of the diluted samples were applied in duplicates and incubated for 1.5 h at 37 °C. The detection antibody (polyclonal anti β-galactosidase from rabbit, PA1-21477, Thermo Scientific, Schwerte, Germany) was applied at a 1:8000 dilution (in blocking buffer) over night at 4 °C. Then, the secondary antibody (Goat Anti Rabbit IgG H&L antibody HRP, Biorbyt Limited, Cambridge, United Kingdom) was applied (1:20,000 dilution in blocking buffer) for 2 h at RT. Detection was achieved by application of 3,3′,5,5′-tetramethylbenzidine (TMB) Substrate Solution (Thermo Scientific, Schwerte, Germany) according to manufacturer’s instructions. The colorimetric change was detected at 450 nm and a standard with purified β-galactosidase was run along at concentrations of 0.05–1.0 µg/mL for quantification.

### Flow cytometry

Flow cytometry was performed using the CyFlow^®^ SL (Partec, Münster, Germany). Samples were stained with 0.75 µM Bis-(1,3-Dibarbituric acid)-trimethine oxanol (DIBAC_4_ [3]) and 3 µM *N*-(3-Triethylammoniumpropyl)-4-(4-(4-(diethylamino)phenyl)butadienyl)pyridinium dibromide (RH414) prior to analysis. The determination of populations was performed according to Langemann et al. [14] and Jechlinger et al. [29]. The particle concentration from populations with positive RH414 staining was used to adjust particle concentrations of whole cells and cellular envelopes for activity assays and ELISA.

### Liposome production

Thin layer rehydration was used to generate liposomes as SUV. 2 mg/mL 1-palmitoyl-2-oleoylphosphatidylcholine (POPC) were diluted in ethanol (99.5 %) in a 5 mL round bottom flask and a thin layer was formed using a rotational evaporator. The lipid was further dried using a vacuum desiccator for 30 min. Then, 2 mL PBS (pH 7.4) were added and film rehydration was achieved by rotation for 30 min at RT. The liposomes were cleared from not incorporated lipid by centrifugation for 6 min at 13,000×*g* and sonicated using a 2 mm tip sonicator for 3 min with an amplitude of 25.3 µm. Then, the concentrated soluble protein containing β-gal-cyt b_5_ was added at a ratio of 1:3 and 1:5 to the liposomes. The liposomes were incubated for 1 h at 30 °C at 250 rpm in a thermal shaker and subsequently washed twice by ultracentrifugation at 125,000×*g* for 1 h, 4 °C. SDS-Page analysis of liposomes was performed according to Sambrook and Russell [27].

### Activity assay

β-Galactosidase activity assays were detected using *ortho*-nitrophenyl-β-galactoside (oNPG). In 1 mL total volume 50 µL of diluted sample were analyzed in 0.1 M potassium phosphate buffer containing 0.124 M 2-mercaptoethanol, 1 mM MgCl_2_, pH 7.0 with 1 mM oNPG at RT. The colorimetric change was detected at 436 nm and the slope was used to calculate the corresponding activity using the molar extinction coefficient ε_oNP_ = 3.51/(mM cm). The activity in permeate from cellular envelope workup using tangential flow filtration and in supernatant from SUV production was measured in order to detect background activity from soluble β-gal-cyt b_5_. The results were corrected accordingly.

## Additional files

10.1186/s12934-015-0371-9 Cellular envelope weight to count correlation. Defined amounts of lyophilized cellular envelopes from *E.* *coli* BL21 (DE3) (black) and *E.* *coli* C41 (DE3) (grey) batch process without immobilized enzymes were analyzed using flow cytometry. The populations with positive signal for RH414 staining were used to calculate a correlation from the linear slope (see equation S1).
10.1186/s12934-015-0371-9 Comparison of β-galactosidase activities without detergent treatment. The activity of equal amounts of β-galactosidase before and after detergent treatment are compared.
10.1186/s12934-015-0371-9 SDS-PAGE from SUV with immobilized β-gal-cyt b_5_. Comparison of [1] purified β-galactosidase with [2] SUV with immobilized β-gal-cyt b_5_ and [3] background from supernatant after liposome workup. Immobilized β-gal-cyt b_5_ and β-galactosidase reference are highlighted. Notably, the reference has no membrane anchor and is consequently smaller.
10.1186/s12934-015-0371-9 Cellular envelope particle count to weight correlation (equation) and sequences of primers encoding the cyt b_5_ anchor.

## Abbreviations

β-gal-cyt b_5_: β-galactosidase from *E.* *coli* K12 fused to the C-terminal hydrophobic sequence of cytochrome b_5_ from rabbit liver
SUV: small unilamellar vesicle
FACS: fluorescence aided cell sorting
ELISA: enzyme linked immunosorbent assay
Cfu: colony forming units
CDW: cell dry weight
