# Supplementary figures and images for: A novel one-step expression and immobilization method for the production of biocatalytic preparations
Source: Microb Cell Fact. 2015 Nov 14;14:180. doi: 10.1186/s12934-015-0371-9 (PMC4650107; doi:10.1186/s12934-015-0371-9)

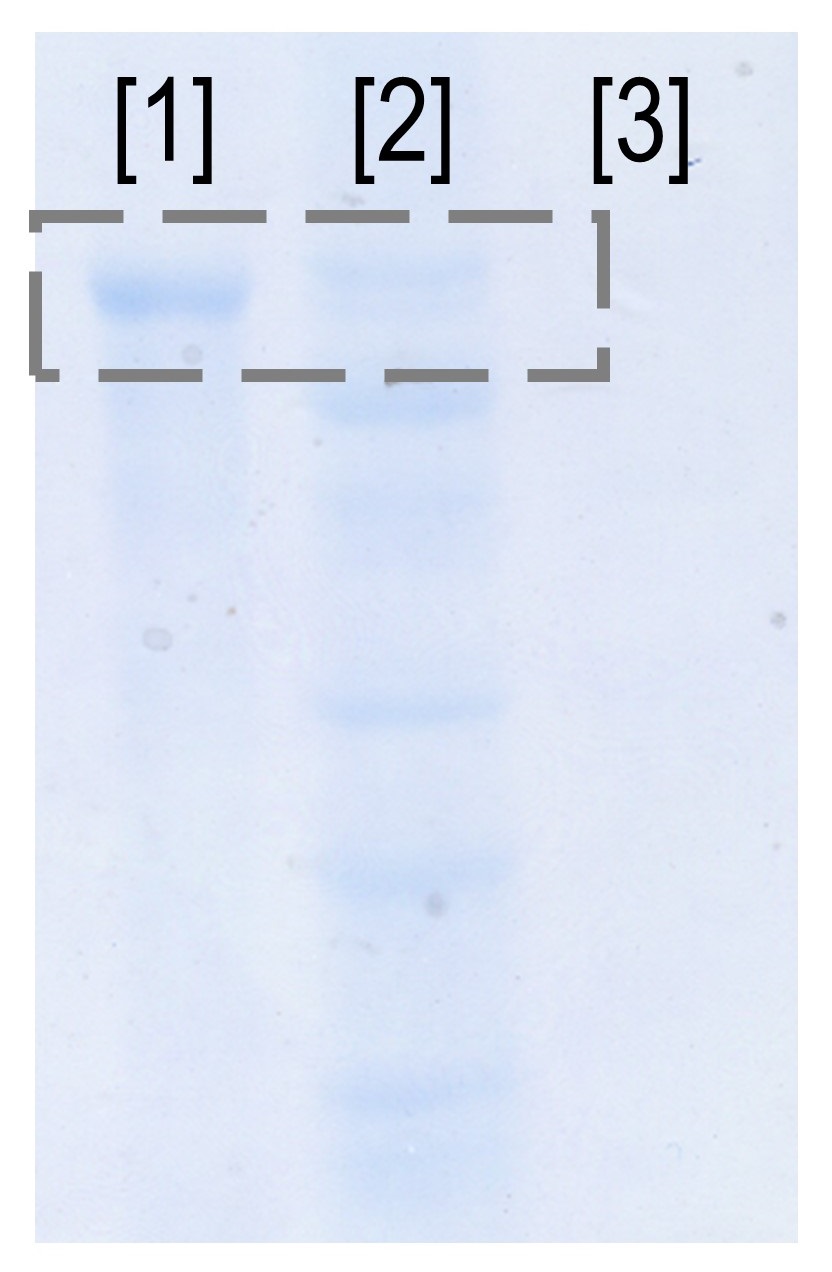

Supplement: Supplementary file 3 — 10.1186/s12934-015-0371-9 SDS-PAGE from SUV with immobilized β-gal-cyt b5. Comparison of [1] purified β-galactosidase with [2] SUV with immobilized β-gal-cyt b5 and [3] background from supernatant after liposome workup. Immobilized β-gal-cyt b5 and β-galactosidase reference are highlighted. Notably, the reference has no membrane anchor and is consequently smaller. [file 12934_2015_371_MOESM3_ESM.jpg]
