# Supplementary material for: A novel one-step expression and immobilization method for the production of biocatalytic preparations
Source: Microb Cell Fact. 2015 Nov 14;14:180. doi: 10.1186/s12934-015-0371-9 (PMC4650107; doi:10.1186/s12934-015-0371-9)
Supplement: Supplementary file 4 — 10.1186/s12934-015-0371-9 Cellular envelope particle count to weight correlation (equation) and sequences of primers encoding the cyt b5 anchor. [file 12934_2015_371_MOESM4_ESM.pdf]

Supplementary information

Cellular envelope particle count to weight correlation

Cellular envelopes without immobilized enzymes for the determination of the cellular envelope to dry weight correlation were obtained using a batch cultivation without induction as described in Langemann et al.. After workup, the cellular envelopes were lyophilized and subsequently used to generate a particle count to weight correlation (see figure S1). The resulting equation is given below.

$$DW = 1.33 \cdot 10^{-9}\ \text{g mL L}^{-1} \cdot Count_{FACS} + 0.92\ \text{g L}^{-1} \qquad \text{Equation S1}$$

with

DW Dry weight concentration of cellular envelopes, g L^{-1}

$Count_{FACS}$ Count of positive fluorescence signals at 590 nm in flow cytometry using RH414 staining, mL^{-1}

Sequences of primers encoding the cyt b_5 anchor

The primers for cloning of the cytochrome b_5 membrane anchor are summarized in table S1. They were ordered as 5'-phosphorylated single strands:

Table S1. Sequences of primers encoding the cyt b_5 anchor

Forward primer	5'-CGCGCCTGAGCAAACCGATGGAAACCCTGATTACCACCGTGGATAGCAATAGCAGCTGGTGGACCAATTGGGTGATTCCGGCGATTAGCGCGCTGATTGTGGCGCTGATGTATCGTCTGTATATGGCGGATGATTAGC-3'
Reverse primer (reverse complement)	5'-GGCCGCTAATCATCCGCCATATACAGACGATACATCAGCGCCACAATCAGCGCGCTAATCGCCGGAATCACCCAATTGGTCCACCAGCTGCTATTGCTATCCACGGTGGTAATCAGGGTTTCCATCGGTTTGCTCAGG-3'

References

Langemann T, Koller VJ, Muhammad A, Kudela P, Mayr UB, Lubitz W. The bacterial ghost platform system: Production and applications. Bioeng Bugs. 2010;1(5):326–36.
