# Supplementary material for: A novel one-step expression and immobilization method for the production of biocatalytic preparations
Source: Microb Cell Fact. 2015 Nov 14;14:180. doi: 10.1186/s12934-015-0371-9 (PMC4650107; doi:10.1186/s12934-015-0371-9)
Supplement: Supplementary file 3 [file 12934_2015_371_MOESM1_ESM.docx]

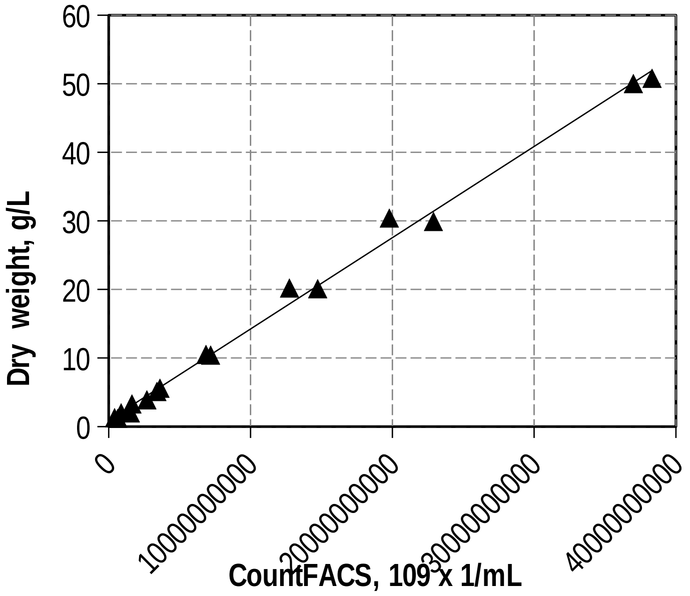
60
50
40
30
20
10
0
Dry weight, g/L
0
10000000000
20000000000
30000000000
40000000000
CountFACS, 109 x 1/mL
