# Supplementary material for: A novel one-step expression and immobilization method for the production of biocatalytic preparations
Source: Microb Cell Fact. 2015 Nov 14;14:180. doi: 10.1186/s12934-015-0371-9 (PMC4650107; doi:10.1186/s12934-015-0371-9)
Supplement: Supplementary file 4 [file 12934_2015_371_MOESM2_ESM.docx]

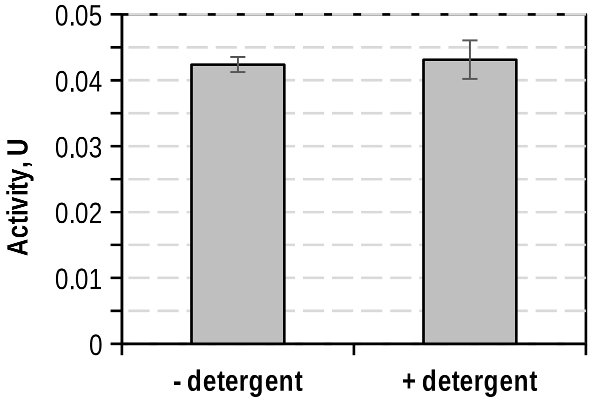

0.05
0.04
0.03
0.02
0.01
0
Activity, U
- detergent
+ detergent
